# 

**Published:** 1828-02

**Authors:** 


### MONTHLY LIST OF MEDICAL BOOKS.

[*Medical Works cannot be entered on this List except a copy be sent for the purpose; the titles of Books having frequently been transmitted to us, as published, which have not appeared for weeks, or even months, after.*]

A General View of the present State of Lunatics, and Lunatic Asylums, in Great Britain and Ireland, and in some other Kingdoms. By Sir Andrew Halliday, M.D. & K.H. Fellow of the Royal Society and College of Physicians of Edinburgh, Licentiate of the Royal College of Physicians of London, &c. &c.—8vo. London, 1828.

Observations on the Mortality and Physical Management of Children. By John Roberton, M.R.C.S. Edinburgh, &c.—London, 1827.

